# Supplementary material for: Turning defence into offence? Intrusion of cladoceran brood chambers by a green alga leads to reproductive failure
Source: R Soc Open Sci. 2020 Sep 16;7(9):200249. doi: 10.1098/rsos.200249 (PMC7540781; doi:10.1098/rsos.200249)
Supplement: Figures [file rsos200249supp1.docx]

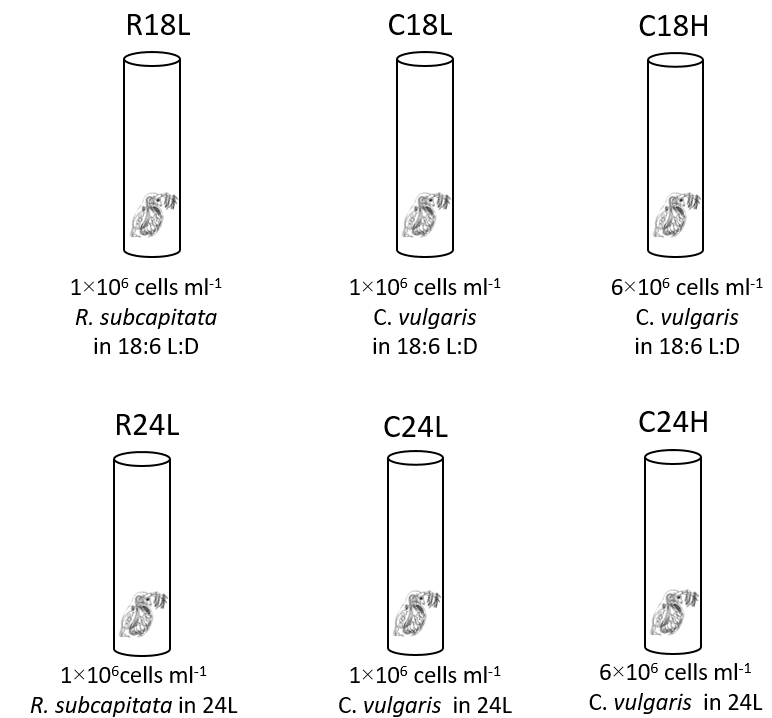
**Supplementary materials**

Fig. S1. Schematic diagram for Experiment 1 treatments: R denotes *Raphidocelis subcapitata*;; C denotes *C. vulgaris*; 18 denotes a 18hrs light:6hrs dark photoperiod; 24 denotes continuous light at 70 µmol photons m^-1^ s^-1^. H and L indicate respectively high and low algae concentration. There were 20 replicates per treatment.


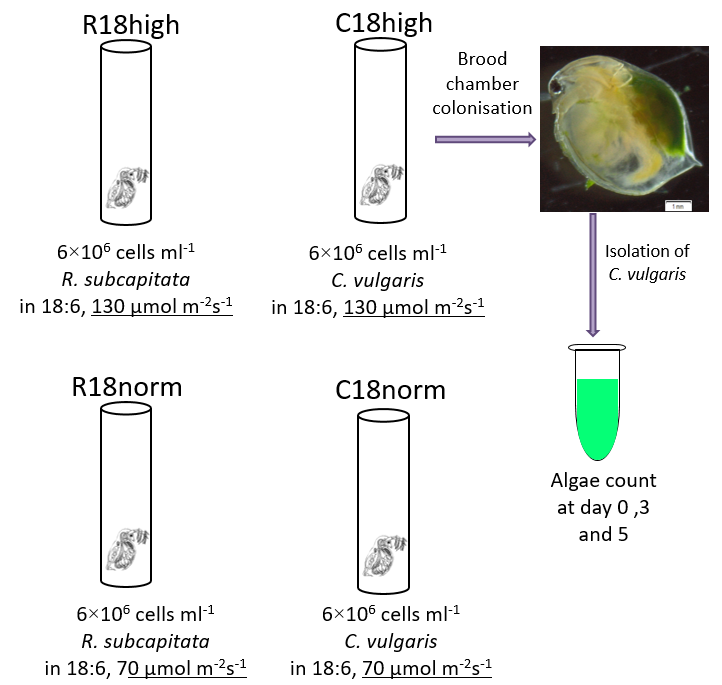


Fig. S2. Schematic diagram for Experiment 2 treatments: R denotes *Raphidocelis subcapitata*; C denotes *Chlorella vulgaris*; 18 denotes a 18hrs light:6hrs dark photoperiod; high denotes a light intensity of 130 µmol photons m^-2^s^-1^ and norm 70 µmol photons m^-2^s^-1^, respectively. There were 20 replicates per treatment.


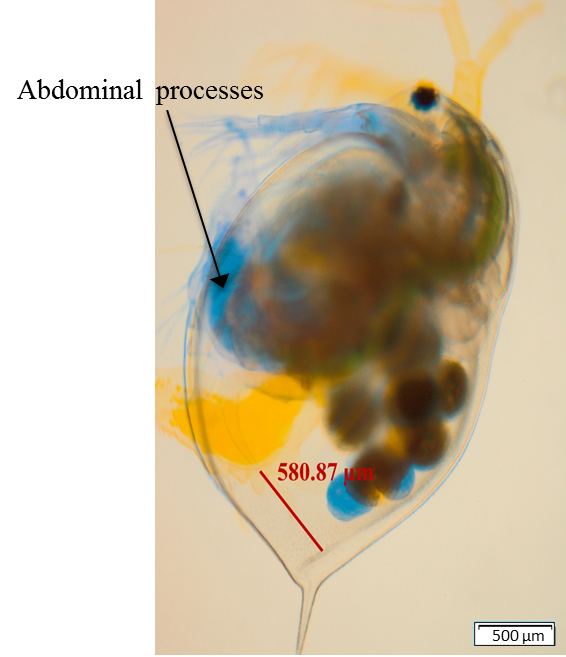


Fig. S3. Example of one of 50 pictures taken to measure the aperture (red line) of *D. magna* abdominal process. *D. magna* opened the abdominal processes while flexing its abdomen. Photo was taken with an Olympus BX43 microscope and a UC30 Olympus Camera.
